# Emotion recognition from multimodal biosignals: supervised and unsupervised machine learning approaches based on EEG and GSR

**DOI:** 10.3389/fpsyg.2026.1835911

**Published:** 2026-07-08

**Authors:** María Consuelo Sáiz-Manzanares, Raúl Marticorena-Sánchez

**Affiliations:** 1 GIR DATAHES, Consolidated Research Unit N.° 348 JCYL, Health Sciences Department, Universidad de Burgos, Burgos, Spain; 2 GIR ADMIRABLE, Consolidated Research Unit N.° 170 JCYL, Computer Engineering Department, Universidad de Burgos, Burgos, Spain

**Keywords:** Electroencephalogram (EEG), emotion recognition, Galvanic skin response (GSR), higher education, instructional psychology, machine learning, multimodal biosignals

## Abstract

**Introduction:**

Artificial intelligence (AI) and machine learning (ML) are increasingly applied in psychology, particularly in educational and clinical settings, to analyse complex behavioural and physiological data. However, evidence regarding the capacity of multimodal physiological signals to characterise cognitive and emotional processes in ecologically valid higher education environments remains limited. This study explored the potential of supervised and unsupervised ML approaches to analyse multimodal physiological responses elicited by emotional avatars in a real educational context.

**Methods:**

A convenience sample of 55 participants, including university students and lecturers, was recruited. After data cleaning, the final sample comprised 48 participants. Electroencephalography (EEG) and galvanic skin response (GSR) signals were recorded while participants observed avatars displaying different emotional states. Event-related potential (ERP)-derived features were extracted from EEG recordings. Supervised learning was conducted using a Random Forest classifier to evaluate the predictive contribution of physiological, subjective (NASA-TLX), and sociodemographic variables in classifying professional category. In addition, unsupervised analyses were performed using k-means clustering and principal component analysis (PCA) to identify latent patterns within the physiological data.

**Results:**

Sociodemographic variables achieved the highest predictive performance, followed by subjective workload measures, whereas physiological features demonstrated more limited discriminative power. Cluster analysis revealed substantial overlap among participant response profiles, and PCA showed limited separation between groups in the reduced-dimensional space. These findings suggest that physiological responses associated with emotional processing are better represented as continuous patterns of variability rather than as clearly differentiated categories.

**Discussion:**

The findings highlight both the opportunities and limitations of applying ML techniques to multimodal physiological data in ecologically valid educational settings. Although physiological measures alone showed limited predictive capacity, they provided complementary information regarding cognitive and emotional processes. Furthermore, the study proposes a dashboard-based framework for the automated integration, analysis, and visualisation of multimodal data, supporting future developments in educational psychology, affective computing, and precision psychology. Overall, the results underscore the need for larger samples and more advanced multimodal modelling strategies to improve the interpretability and predictive value of physiological signals in real-world contexts.

## 1 Introduction

Artificial intelligence (AI) refers to the development of systems that can perform tasks that typically require human intelligence, such as reasoning, decision-making, and pattern recognition (Nilsson, 1998; Russell and Norvig, 2021). Within this field, machine learning (ML) involves algorithms that learn from data to improve performance in specific tasks (Mitchell, 1997). More recently, Generative Artificial Intelligence (GAI) has built on these capabilities by enabling the generation of new content based on learned patterns. GAI is increasingly being applied in areas such as education, psychology, and simulation environments (Chen et al., 2024; García-Peñalvo, 2023, 2024; Prégent et al., 2025; Sengar et al., 2025).

The application of AI, ML, and GAI in psychology has evolved from a theoretical possibility to an established area of research. These technologies are currently used for assisted diagnosis through predictive models, behavioural modelling and computational representations of cognitive processes, as well as for analysing large amounts of data in order to identify risk factors and patterns relating to mental health and wellbeing (Cruz-González et al., 2025; Gomes et al., 2023; Russell and Norvig, 2021). Additionally, ML techniques can identify complex relationships across multimodal data sources, such as physiological, behavioural, and contextual variables. This facilitates applications such as neuroimaging analysis, personalised psychological assessment and the detection of behavioural patterns derived from digital interactions (Chen et al., 2022; Mitchell, 1997; Tejavibulya et al., 2022). In this context, these approaches are particularly relevant in settings characterised by high-dimensional data and relatively small sample sizes, where robust modelling strategies and feature selection techniques are required to reduce overfitting and improve model interpretability, in line with established methodological recommendations (Hastie et al., 2009; James et al., 2021).

Within this context, pedagogical avatars have been conceptualised as emotional and metacognitive scaffolding devices that support learners in interpreting information and regulating their activity in digital environments. In multimodal learning contexts, these agents can modulate physiological arousal and influence affective processing, thereby contributing to self-regulated learning processes (Mudrick et al., 2019; Zheng et al., 2023). Recent research has also emphasised their potential in stealth assessment and adaptive learning environments, particularly in game-based learning and complex problem-solving scenarios such as medical diagnosis (Cloude et al., 2025; Dever et al., 2025). Concurrently, AI and ML techniques have increasingly been applied to analyse learning processes in e-learning environments, particularly in the study of self-regulated learning (Azevedo, 2020). Approaches such as process mining can identify behavioural patterns and learning strategies from interaction data, enabling a more detailed assessment of learning processes (Cerezo et al., 2020).

From a methodological perspective, ML techniques can be classified as either supervised or unsupervised. Supervised methods are used when labelled data are available, typically for classification and regression tasks (Montgomery et al., 2012). In contrast, unsupervised methods focus on identifying latent structures and patterns without predefined labels (James et al., 2021). These techniques are particularly relevant in psychological research, as they enable the modelling of relationships between physiological signals [e.g. Electroencephalography (EEG) and Galvanic Skin Response (GSR)], behavioural indicators, and psychological constructs, such as cognitive load, emotional states, and learning profiles (Fleming et al., 2023; Gao et al., 2023; Gevins and Smith, 2003; Kreienkamp et al., 2025; Mkrtchian et al., 2021). Beyond physiological signals, multimodal emotion recognition approaches incorporate additional data modalities such as facial expressions and body gestures. These modalities provide Supplementary information about emotional states and have been successfully analysed using ML techniques in recent studies (e.g., Xia et al., 2026; Xia and Peng, 2020). Integrating these different data sources contributes to a more comprehensive understanding of human emotion in AI-based psychological applications. However, applying ML to multimodal biometric data such as EEG, GSR and eye-tracking requires systematic procedures for data extraction, cleaning, synchronisation and transformation prior to analysis (Fu et al., 2024; García et al., 2015; Gong et al., 2023; Jamal et al., 2023; Nolte et al., 2025; Sáiz-Manzanares et al., 2026). These steps are particularly relevant in contexts characterised by high-dimensional data and relatively small sample sizes, as is often the case in applied research in the health sciences and education. Under these conditions, feature selection, dimensionality reduction, and robust, interpretable modelling are essential for meaningful and reliable analysis (Liu et al., 2002; Ramadan et al., 2024; Tarnowski et al., 2018).

Below, we describe the most representative ML algorithms and their application to the analysis of learning processes monitored through integrated multichannel devices, such as eye-tracking, EEG and GSR. The contributions of AI, ML and GAI to psychology are summarised in Table 1.

**Table 1:** Contributions of AI, ML, and GAI to psychology.

Approach	Type of contribution to psychology	Example
AI	Cognitive modelling, simulation, and Rule-based reasoning	Expert systems for clinical assessment and learning evaluation
ML	Prediction, classification, and analysis of complex patterns	Risk detection for depression and learning difficulties using EEG or social media data
GAI	Content generation and simulation of psychological and educational processes	Chatbots for cognitive behavioural therapy and self-regulated learning systems

### 1.1 Physiological signals in emotion recognition

The application of ML to multimodal biometric data, such as EEG, GSR and eye-tracking, requires systematic procedures for data extraction, cleaning, synchronisation and transformation prior to analysis (Fu et al., 2024; García et al., 2015; Gong et al., 2023; Jamal et al., 2023; Nolte et al., 2025; Sáiz-Manzanares et al., 2026). These steps are particularly relevant in contexts characterised by high-dimensional data and relatively small sample sizes, as is often the case in applied research in the health sciences and education. In such scenarios, feature selection, dimensionality reduction and the utilisation of robust, interpretable models are crucial for ensuring meaningful, reliable analysis (Liu et al., 2002; Ramadan et al., 2024; Tarnowski et al., 2018). While several benchmark datasets (e.g., DEAP, AMIGOS, and SEED) have advanced emotion recognition models (Koelstra et al., 2012; Miranda-Correa et al., 2018; Zheng and Lu, 2015), these datasets are usually obtained in controlled laboratory settings, which may not reflect the complexity of real-world educational environments. This reduces their ecological validity and restricts their applicability in instructional contexts (Zheng et al., 2014; Jones, 2017). Therefore, exploratory studies are needed to examine how multimodal physiological data can be collected, processed, and interpreted in authentic learning settings. This is consistent with the broader Big Data perspective in cognitive science, which emphasises analysing complex, high-dimensional datasets derived from real-world contexts to improve our understanding of cognitive and behavioural processes (Jones, 2017). This approach is particularly relevant when ecological validity and practical applicability are prioritised over model optimisation (Zheng et al., 2014; Fu et al., 2024). The most representative ML algorithms and their application to the analysis of learning processes monitored through integrated multichannel devices, such as eye-tracking, EEG, and GSR, are described below.

### 1.2 Applying machine learning approaches to multimodal data analysis

ML techniques can be broadly classified into supervised and unsupervised approaches, depending on the availability of labelled data. Supervised learning is typically applied to classification and prediction tasks, enabling the modelling of relationships between physiological, behavioural, and contextual variables and specific psychological outcomes. In contrast, unsupervised learning focuses on identifying latent structures and patterns in data without predefined labels, making it particularly suitable for exploratory analyses in complex and high-dimensional datasets (Gao et al., 2023; Kreienkamp et al., 2025; Fleming et al., 2023; Mkrtchian et al., 2021).

In the field of psychology, these approaches have been widely applied to the analysis of multimodal biometric data, including EEG, GSR, and eye-tracking. The processing of such data requires systematic preprocessing procedures—such as temporal synchronisation, noise and artefact removal, normalisation, and feature extraction—to ensure data quality and comparability across modalities (García et al., 2015). These steps are particularly relevant in applied research contexts characterised by small sample sizes and high-dimensional feature spaces, where feature selection and dimensionality reduction techniques are often required to improve interpretability and robustness (Liu et al., 2002; Ramadan et al., 2024; Sáiz-Manzanares et al., 2021; Tarnowski et al., 2018).

Within this framework, the application of ML techniques to multimodal physiological data enables the identification of patterns related to cognitive and emotional processes, as well as the exploration of relationships between variables across different data channels. This is especially relevant in exploratory studies conducted in real-world educational settings, where the objective is not only predictive performance but also interpretability and practical applicability.

### 1.3 Dashboard design for multimodal data in learning environments

To enhance the practical applicability of results derived from the use of AI, ML and GAI in conjunction with integrated multichannel signal acquisition devices, dashboard design emerges as a key component. These systems can be applied across different areas of psychology, particularly in educational and clinical contexts.

Dashboards are interactive visual interfaces that integrate, process, and represent large volumes of data in a structured, decision-oriented manner. They facilitate the interpretation of complex information by providing dynamic visualisations that support the identification of patterns, trends and relationships between variables, thereby reducing cognitive load (Cabral et al., 2025; Martínez et al., 2024; Susnjak et al., 2022; Verbert et al., 2024). In multimodal data collection contexts, dashboards facilitate the integration of diverse data sources, such as eye-tracking, EEG and GSR, synchronised via shared timestamps. This integration provides a comprehensive view of user behaviour, combining visual, neurophysiological, and psychophysiological information within a single analytical environment (Jamal et al., 2023; Nolte et al., 2025; Sáiz-Manzanares et al., 2024). In educational and psychological contexts, these tools facilitate the integration of physiological and behavioural data, supporting exploratory analysis and decision-making processes.

From a functional and technical perspective, dashboard systems usually comprise multiple analytical layers, including data acquisition, preprocessing, modelling and visualisation. This allows raw multichannel data to be transformed into interpretable outputs. Integrating ML techniques enables the automated analysis of large datasets, including supervised approaches for prediction and classification, as well as unsupervised approaches for pattern detection (Mella Norambuena et al., 2022; Susnjak et al., 2022; Verbert et al., 2024).

In this context, incorporating Generative AI extends dashboards’ capabilities further by enabling automated summaries, natural language interaction, adaptive feedback and decision support. From an exploratory perspective, these systems can act as an interpretative layer that facilitates understanding of complex model outputs, complementing rather than replacing expert judgement (Jin et al., 2024; Doshi-Velez and Kim, 2017; Pinargote et al., 2024). Building on this framework and the state of the art previously outlined, the present study proposes an exploratory application of ML techniques for processing multimodal physiological data obtained from EEG and GSR devices within an integrated analysis environment. In parallel, it proposes a dashboard design to automate data collection, processing and visualisation. Dashboards provide interactive environments that facilitate the interpretation of large datasets by representing relationships between variables, trends and patterns in a structured, accessible way (Cabral et al., 2025; Martínez et al., 2024; Mella Norambuena et al., 2022; Susnjak et al., 2022; Verbert et al., 2024). In this sense, dashboards act as a bridge between complex analytical processes and their practical application in real-world contexts.

Dashboards are a particularly suitable tool in exploratory studies, as they enable the integration of multiple data sources, the exploration of relationships across variables, and the iterative refinement of analytical models in real-world contexts. Recent developments in multimodal learning analytics systems further demonstrate the potential of dashboard-based approaches to synchronise, visualise and interpret physiological and behavioural data collected in authentic learning environments (Becerra et al., 2023; Hong et al., 2025; Kaliisa et al., 2023).

Specifically, the objectives of this study were: (1) to explore the application of ML techniques for the pre-processing and analysis of multimodal data in real-world educational contexts, and (2) to explore the potential of supervised and unsupervised approaches for processing physiological, subjective and sociodemographic data. The study also aimed to propose a dashboard design for integrating and visualising these data (Sáiz-Manzanares et al., 2025).

## 2 Method

### 2.1 Participants

A convenience sample of 55 members of the academic community from the University of Burgos and the University of Valladolid was recruited. This included 26 undergraduate and master’s students of Health Sciences, 16 early-career lecturers with less than 4 years’ teaching experience and 13 experienced lecturers with more than 10 years’ experience. All participants were either enrolled in or teaching within Health Sciences programmes.

Seven participants were excluded due to recording errors identified during the acquisition phase. The final sample therefore consisted of 48 participants: 22 students (14 women and eight men), 16 early-career lecturers (10 women and six men) and 10 experienced lecturers (five women and five men). Table 2 shows the distribution of sociodemographic characteristics for the initial and final samples.

**Table 2:** Sociodemographic characteristics of the sample.

Group	n (total)	Women *n* (%)	Men n (%)	Age range (years)
Undergraduate students	13	8 (61.5%)	5 (38.5%)	20–30
Master’s students	13	6 (46.2%)	7 (53.8%)	20–30
Early-career lecturers	16	10 (62.5%)	6 (37.5%)	20–41
Experienced lecturers	13	6 (46.2%)	7 (53.8%)	31–52
Total (initial)	55	30 (54.5%)	25 (45.5%)	–
Total (final)	48	29 (60.4%)	19 (39.6%)	–

### 2.2 Instruments

**Figure 1:**
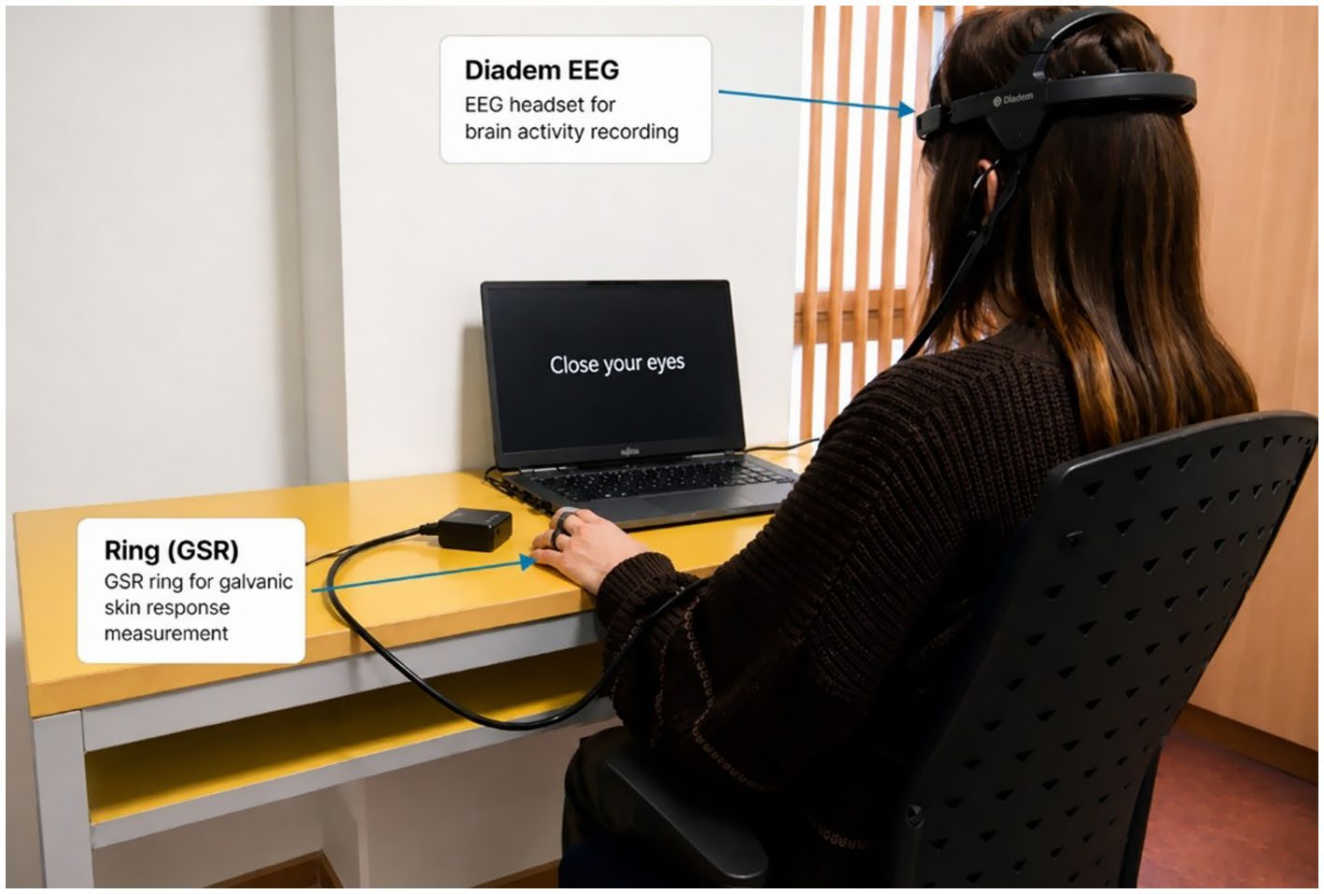
Multichannel integrated signal acquisition setup. The setup includes an EEG headset and a GSR sensor for multimodal physiological data recording.

**Figure 2:**
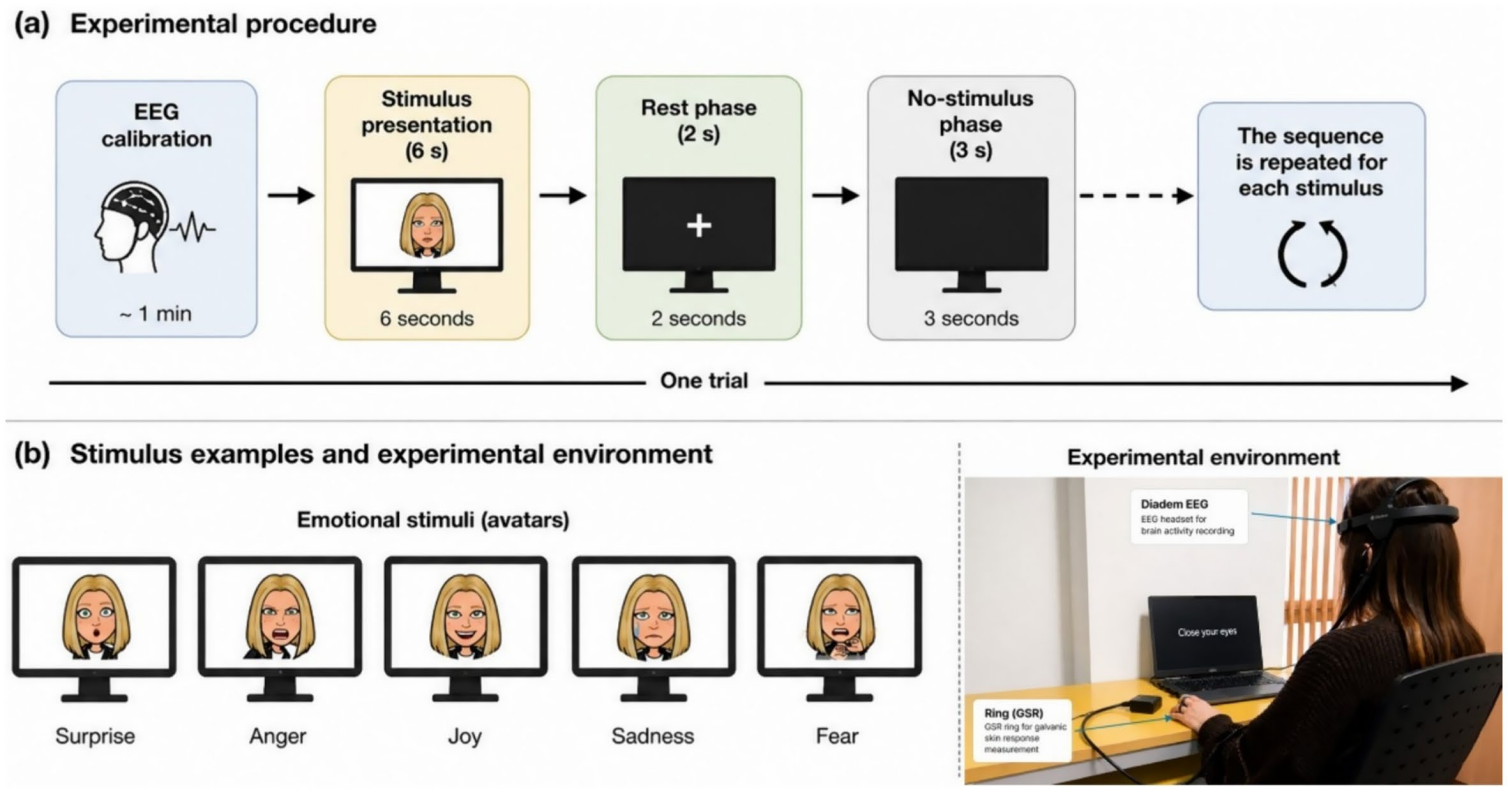
Experimental procedure, stimulus examples, and environment. **(a)** Experimental procedure. Each trial began with EEG calibration (~1 min), followed by stimulus presentation (6 s), a rest phase (2 s), and a no-stimulus phase (3 s). The sequence was repeated for each stimulus. **(b)** Emotional stimuli (avatars representing surprise, anger, joy, sadness, and fear) and experimental environment. EEG and galvanic skin response (GSR) signals were recorded while participants observed the stimuli. The emotional stimuli were derived from the dataset Avatars: emotion recognition (Sáiz-Manzanares, 2024), which comprises computer-generated images created using the Bitmoji platform to depict different emotional states (Gong et al., 2023). The material is publicly available through the University of Burgos Institutional Repository (RIUBU).

Multichannel data acquisition system. A multichannel integrated system was used to record physiological signals. EEG signals were recorded using a Bitbrain headset (sampling frequency: 256 Hz), and GSR was measured using a Bitbrain GSR ring (sampling frequency: 32 Hz). All devices were synchronised via shared timestamps to ensure temporal alignment across modalities (Figures 1, 2).

### 2.3 Procedure

The inclusion criteria were: (a) being an undergraduate or master’s student in Health Sciences, or a lecturer teaching in these programmes; and (b) voluntary participation with informed consent. Exclusion criteria included: (a) incomplete or invalid physiological recordings due to acquisition errors; and (b) self-reported neurological or psychological conditions that could interfere with signal acquisition.

Ethical approval was obtained from the Bioethics Committee of the University of Burgos (reference: IO 5/2024, 21/01/2025). A convenience sampling strategy was applied based on access to students and academic staff from the University of Burgos and the University of Valladolid. All participants provided written informed consent prior to participation.

The experimental sessions were conducted individually under controlled conditions, with standardised lighting and temperature, and in environments free from distracting stimuli. Each session lasted approximately 45 min. (Hart and Staveland, 1988). Prior to the experimental phase, the NASA-TLX questionnaire was administered individually to assess perceived cognitive workload.

During the experimental phase, integrated EEG and GSR signals were recorded using synchronised devices connected via Bluetooth, enabling multimodal data acquisition through shared timestamps. No personally identifiable data were recorded; instead, each participant was assigned an anonymous code. Sociodemographic variables, including age, gender, and professional category, were also collected. The experimental sequence followed a fixed order for all participants to ensure consistency in data collection. Although no randomisation or counterbalancing (e.g., Latin Square design) was implemented, washout periods were included between phases to reduce potential carryover effects. This aspect is considered a limitation of the study.

The data acquisition procedure was structured into the following phases:

- Familiarisation phase (20 s): participants were introduced to the task and calibration procedure. After each stimulus, the word “rest” appeared on the screen, indicating that they should relax.
- Washout phase 1: participants closed their eyes to reach a resting state, allowing physiological responses to stabilise and establishing a baseline.
- Open-eyes phase: resting state with eyes open.
- Calibration phase: presentation of stimuli to determine individual physiological response ranges and enable normalisation across participants.
- Washout phase 2: resting block between calibration and test phases.
- Test phase: presentation of emotional stimuli, each displayed for 6 s.
- SurveyTest: participants classified stimuli as negative, neutral, or positive to estimate emotional valence.
- SurveyCalib: analogous classification applied to calibration stimuli.

After the experimental phase, raw data corresponding to the test phase were extracted from the EEG and GSR devices together with synchronised timestamps. Subsequent preprocessing and feature extraction were performed, following established procedures for EEG signal processing, including filtering, artefact removal, and spectral analysis (Sáiz-Manzanares et al., 2026). For EEG data, mean values of frequency bands (delta, theta, alpha, beta, and low gamma) were computed for each stimulus, along with brain topography representations, following standard approaches in cognitive load assessment using EEG signals (Sáiz-Manzanares et al., 2026). In parallel, for GSR data, features such as amplitude, onset index, peak index, and peak latency were extracted. These measures reflect sympathetic nervous system activity and allow differentiation between tonic (baseline) and phasic components of electrodermal activity.

**Figure 3:**
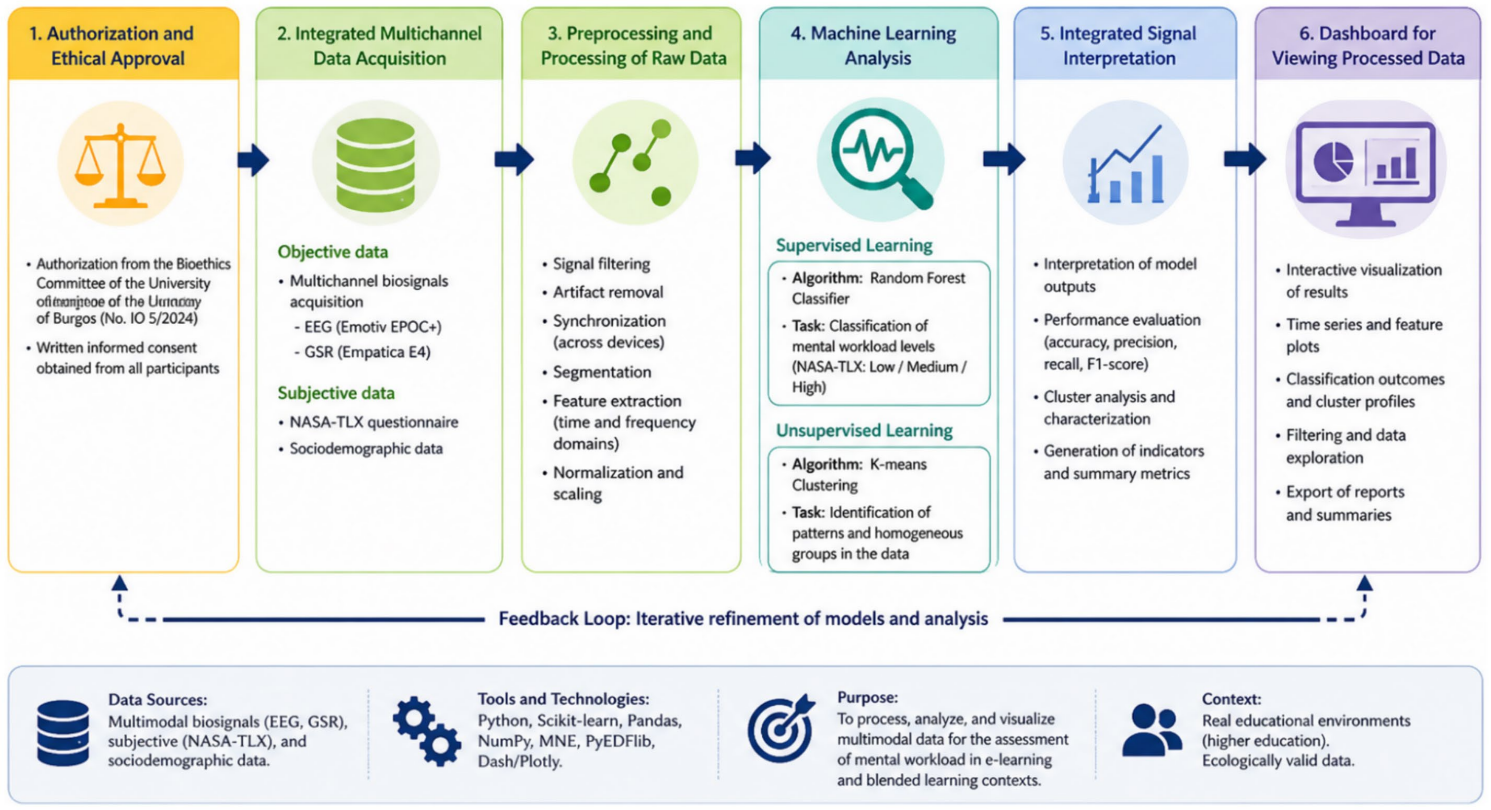
Overview of the proposed pipeline for multimodal data processing, ML analysis, and dashboard integration.

Finally, a dashboard was developed for the processing, analysis, and visualisation of the processed data. A schematic representation of the procedure is shown in Figure 3.

### 2.4 Data analysis

An exploratory study with a quantitative approach was conducted to illustrate the potential application of AI techniques in real educational contexts. The study aimed to identify preliminary patterns in physiological and behavioural data obtained through EEG and GSR, using supervised and unsupervised ML approaches.

The data analysis followed the workflow described in Figure 3. The raw multimodal data (EEG and GSR) were subjected to a preprocessing pipeline that included temporal synchronisation of signals via timestamps, removal of artefacts and invalid segments, normalisation and standardisation of variables, and segmentation into time windows aligned with the different experimental phases.

From the processed signals, modality-specific features were extracted, including temporal metrics and descriptive statistical measures derived from both EEG (e.g., frequency bands and ERP-related features) and GSR (e.g., phasic responses and SCRs-related metrics). Dimensionality reduction techniques, including PCA-based transformation, were subsequently applied to minimise redundancy and improve model stability.

Finally, the resulting feature set was used as input for ML analysis, including supervised classification using a Random Forest classifier and unsupervised pattern detection using k-means clustering, as part of the integrated analytical pipeline.

Within the supervised learning framework, relationships between physiological, subjective, and sociodemographic variables and the target variable (professional category) were explored using classification models. The dataset was partitioned into training and testing subsets using a stratified hold-out strategy to preserve class distribution. Model performance was evaluated using accuracy, balanced accuracy, and macro-averaged F1-score. These metrics were selected to account for potential class imbalance and to provide a more robust assessment of classification performance across categories. Given the exploratory nature of the study and the limited sample size, results were interpreted cautiously as indicative rather than definitive. The final dataset comprised 48 instances and 64 features, representing a small-sample, high-dimensional scenario (small n, large p). Accordingly, algorithms with greater robustness to overfitting, stability under limited sample conditions, and higher interpretability were prioritised. Random Forest was selected as the supervised approach due to its robustness to correlated predictors and its ability to provide feature importance estimates (Breiman, 2001), while k-means clustering combined with PCA-based dimensionality reduction was employed for exploratory unsupervised analysis (Lloyd, 1982; MacQueen, 1967). More variance-sensitive or distance-dependent methods, such as k-nearest neighbours (KNN), were not considered appropriate under these conditions because of their susceptibility to instability and overfitting in small-sample settings (Murphy, 2012).

Given the exploratory nature of the study and the characteristics of the dataset, the analysis focused on descriptive and model-based approaches rather than formal predictive optimisation. This approach is appropriate for hypothesis generation but limits the generalisability of the findings.

### 2.5 Ethical considerations

The study received ethical approval from the Bioethics Committee of the University of Burgos (Report No. IO 5/2024, 21 January 2025) and was conducted in accordance with the ethical principles outlined in the Declaration of Helsinki. All participants provided written informed consent prior to participation, and all data were anonymised through the assignment of non-identifiable alphanumeric codes.

## 3 Results

### 3.1 Multimodal data acquisition and processing model

Raw data were extracted from physiological signal recordings, specifically EEG and GSR. These data were stored in CSV format and subsequently pre-processed and analysed using the following open-source libraries: MNE-Python was used for EEG signal processing and NeuroKit2 for GSR/EDA analysis.

For the EEG data, a standard montage based on the international 10–20 system was used, adapted to the available sensors. A total of 12 channels were included: AF7, Fp1, Fp2, AF8, F3, F4, P3, P4, PO7, O1, O2 and PO8. Preprocessing involved applying Independent Component Analysis (ICA) to remove artefacts caused by physiology (e.g., eye and muscle movement) and movement-related noise. Subsequently, a band-pass filter between 1 and 45 Hz was applied (Hyvärinen et al., 2001). These preprocessing steps follow the established protocols for EEG signal analysis in cognitive load assessment (Sáiz-Manzanares et al., 2026). The preprocessed EEG signals were then segmented according to experimental events corresponding to stimulus presentation. For each segment, power spectral density (PSD) and relative band power were computed for the main frequency bands (delta, theta, alpha, beta, and low gamma). In addition, brain topography representations were generated.

For the GSR/EDA signals, preprocessing included decomposing the signals into their tonic and phasic components using NeuroKit2, in accordance with the standard procedures for analysing electrodermal activity. The tonic component reflects slow-varying baseline activity, while the phasic component captures rapid, event-related responses. Skin conductance responses (SCRs) were automatically detected from the phasic signal based on amplitude and temporal criteria. The extracted features included onset, peak, amplitude and latency, which are commonly used as indicators of sympathetic nervous system activation (Tarnowski et al., 2018; Ramadan et al., 2024).

**Figure 4:**
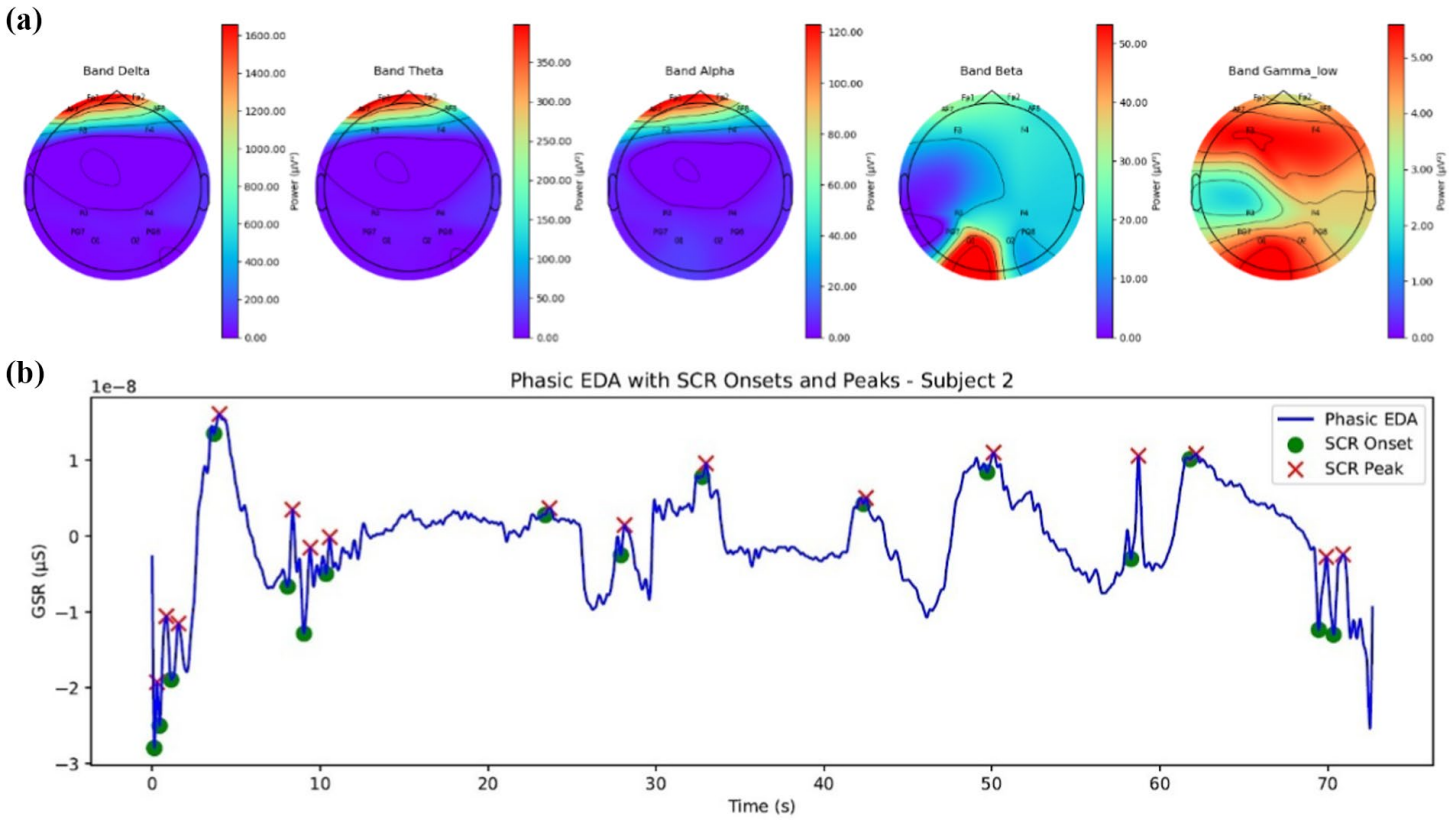
Representative examples of multimodal physiological signal processing outputs. **(a)** Topographical distribution of EEG power across frequency bands (Delta, Theta, Alpha, Beta, and Low Gamma), representing spatial patterns of neural activity derived from processed EEG recordings rather than raw temporal waveforms. **(b)** GSR/EDA signal processing illustrating the phasic component of the electrodermal activity signal, including detected skin conductance responses (SCRs), onset points, and peak detection. Additional examples are provided in the Supplementary material.

These steps enabled the construction of a multimodal dataset integrating physiological, behavioural, and contextual variables for subsequent analysis. Figure 4 shows examples of the preprocessing of raw EEG and GSR data. Additional examples are provided in the Supplementary material. All code used in this study is available on GitHub (see the Data Availability Statement).

### 3.2 Supervised learning results

A Random Forest classifier was applied to predict professional category from physiological, subjective, and sociodemographic variables. Model performance was evaluated using accuracy, balanced accuracy, and macro-averaged F1-score. The global model achieved an accuracy of 0.73, a balanced accuracy of 0.64, and a macro F1-score of 0.68, indicating a moderate overall discriminative capacity. However, performance varied across classes, with the model showing high recall for class 1, moderate performance for class 2, and reduced sensitivity for class 3.

To examine the contribution of different variable groups, three independent models were evaluated (see Table 3). Model A (sociodemographic variables) showed limited predictive performance, with an accuracy of 0.53, a balanced accuracy of 0.59, and a macro F1-score of 0.55. This suggests that basic sociodemographic variables alone are not sufficient to reliably discriminate between professional categories. Model B (physiological variables) also showed low performance, with an accuracy of 0.53, a balanced accuracy of 0.42, and a macro F1-score of 0.38. Notably, the model failed to correctly classify instances of class 3, suggesting that physiological signals alone are insufficient to capture category-specific differences. Model C (subjective variables; NASA-TLX) achieved comparatively better performance, with an accuracy of 0.67, a balanced accuracy of 0.58, and a macro F1-score of 0.58. These results suggest that subjective workload measures provide more discriminative information than physiological or basic sociodemographic variables.

**Table 3:** Performance of classification models based on accuracy, balanced accuracy, and macro F1-score across different groups of variables.

Model	Type of variables	Accuracy	Balanced accuracy	Macro F1-score
Global	All variables	0.73	0.64	0.68
Model A	Sociodemographic variables	0.53	0.59	0.55
Model B	Physiological variables (EEG and GSR)	0.53	0.42	0.38
Model C	Subjective metrics (NASA-TLX)	0.67	0.58	0.58

The global model includes all variables, while Models A, B, and C correspond to sociodemographic, physiological, and subjective (NASA-TLX) variables, respectively.

**Figure 5:**
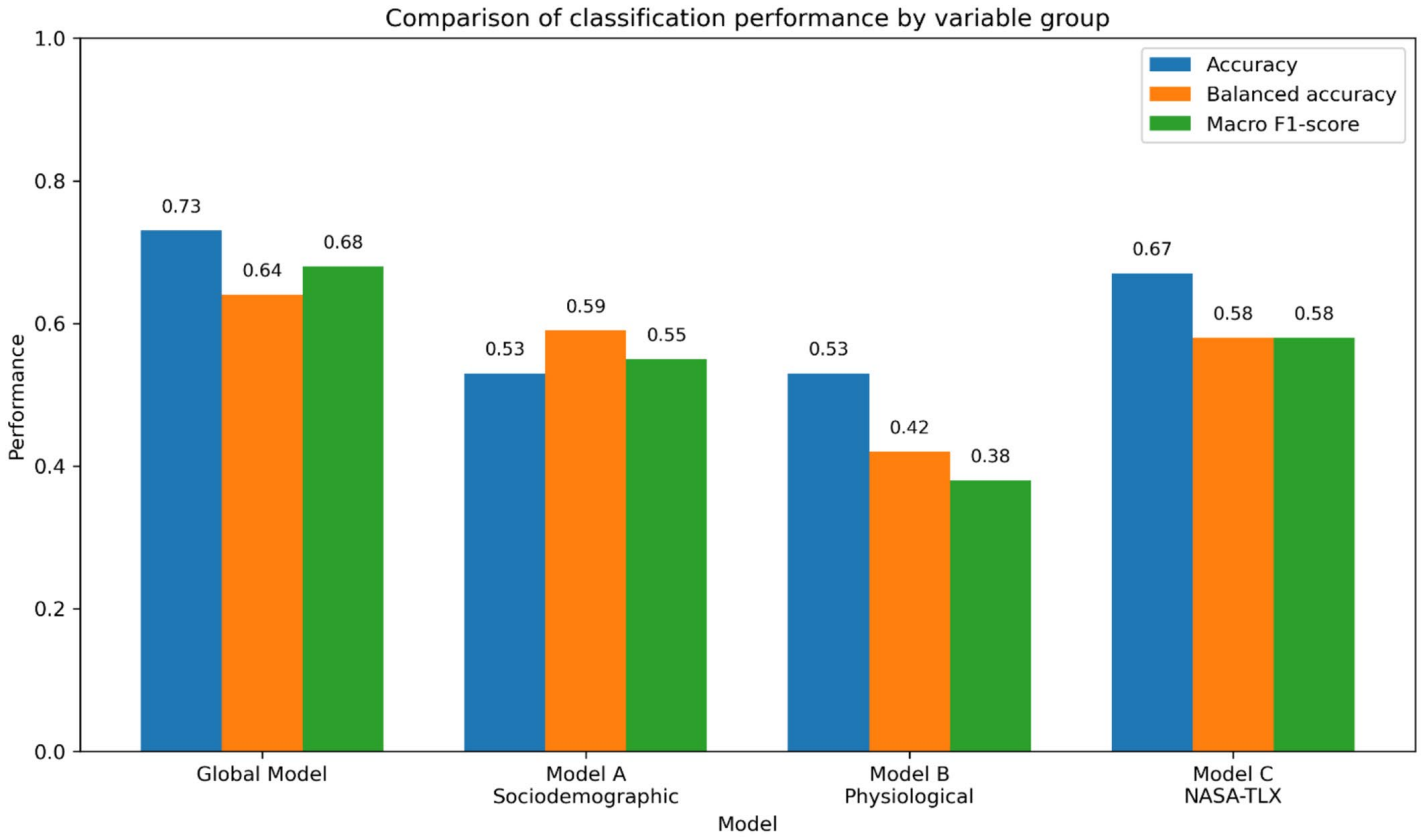
Comparison of model performance based on accuracy, balanced accuracy, and macro F1-score.

As shown in Figure 5, the global model outperformed all individual models, highlighting the benefit of integrating multiple data sources. Although physiological variables alone showed limited classification performance, they contributed to the overall predictive capacity when combined with subjective and sociodemographic predictors. These findings support a multimodal approach for modelling professional category, where complementary information from different domains improves classification performance.

**Figure 6:**
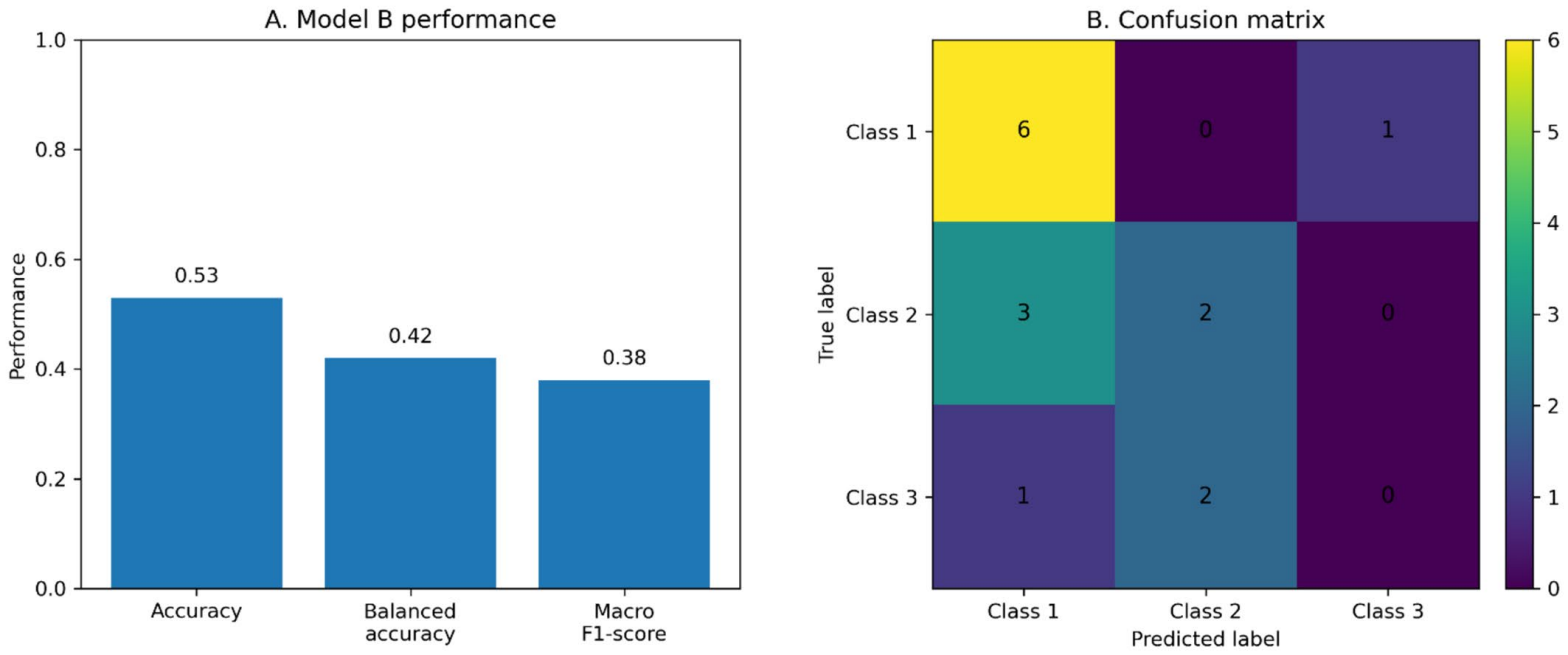
Classification performance of the physiological model (Model B). **(A)** Performance metrics including accuracy, balanced accuracy, and macro-averaged F1-score. **(B)** Confusion matrix showing the distribution of predicted versus true classes across the three professional categories.

As shown in Figure 6A, the model exhibited low performance across all evaluation metrics. This limitation is further illustrated by the confusion matrix (see Figure 6B), which indicates a strong bias towards class 1, along with substantial misclassification of class 2 and a failure to correctly identify instances of class 3.

**Figure 7:**
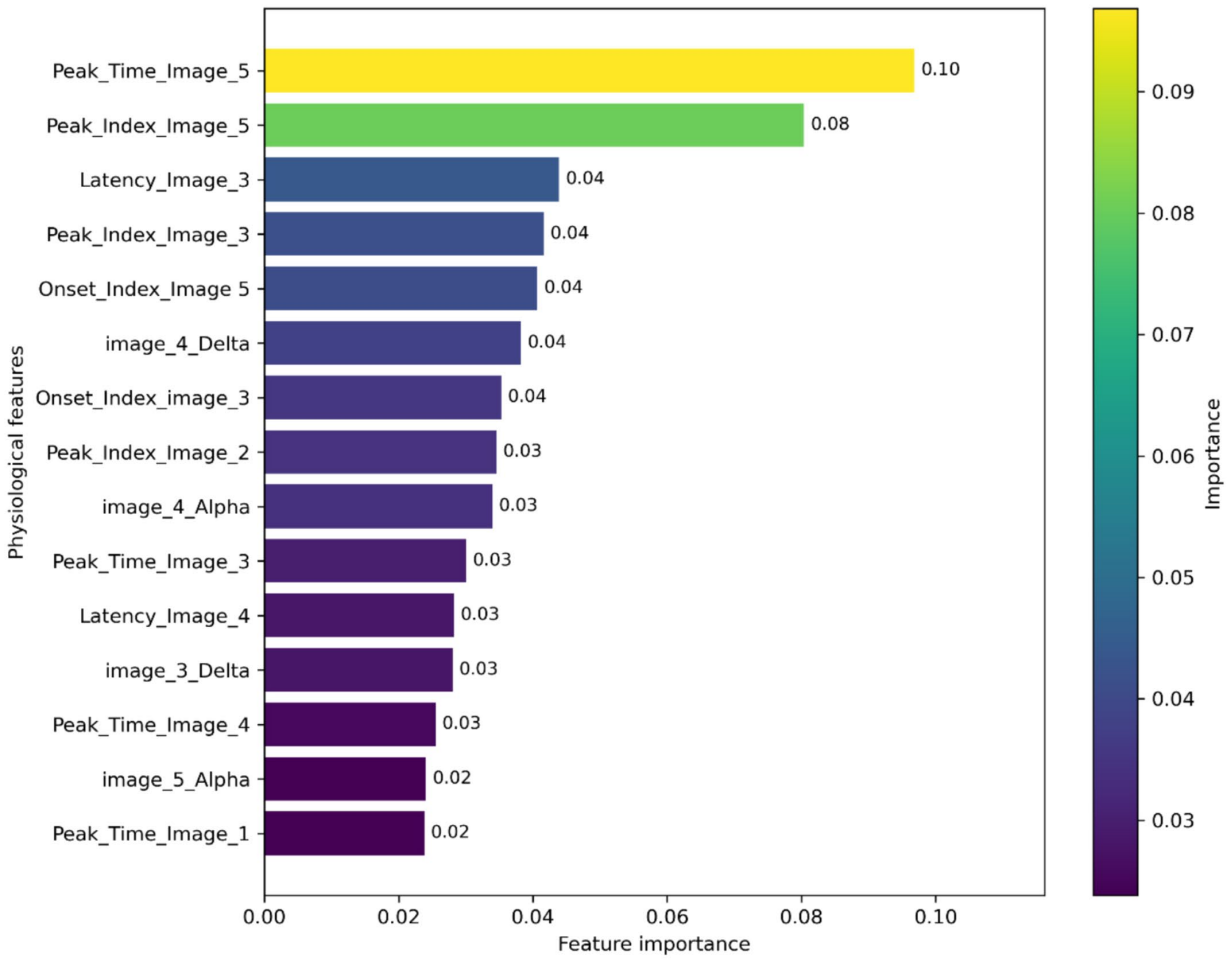
Feature importance of physiological features.

Feature importance analysis in the global model (see Figure 7) indicated that prediction was driven by the combined contribution of multiple variable types. In particular, sociodemographic variables (e.g., age range), selected subjective workload measures (mental demands, effort, and physical demands), and specific physiological indicators contributed to model performance. Among the physiological features, temporally derived metrics—such as peak time, peak index, and latency, particularly from image-based responses—showed the highest relevance. In contrast, frequency-based EEG band features (e.g., delta and alpha) exhibited comparatively lower contributions. These findings suggest that temporally derived physiological markers may contain more discriminative information than frequency-based EEG features. However, their predictive capacity remains limited when considered in isolation.

Overall, the results indicate that no single group of variables is sufficient to accurately predict professional category. Instead, optimal performance arises from the integration of sociodemographic, subjective, and physiological information, supporting a multimodal approach.

### 3.3 Unsupervised learning results (clustering)

**Figure 8:**
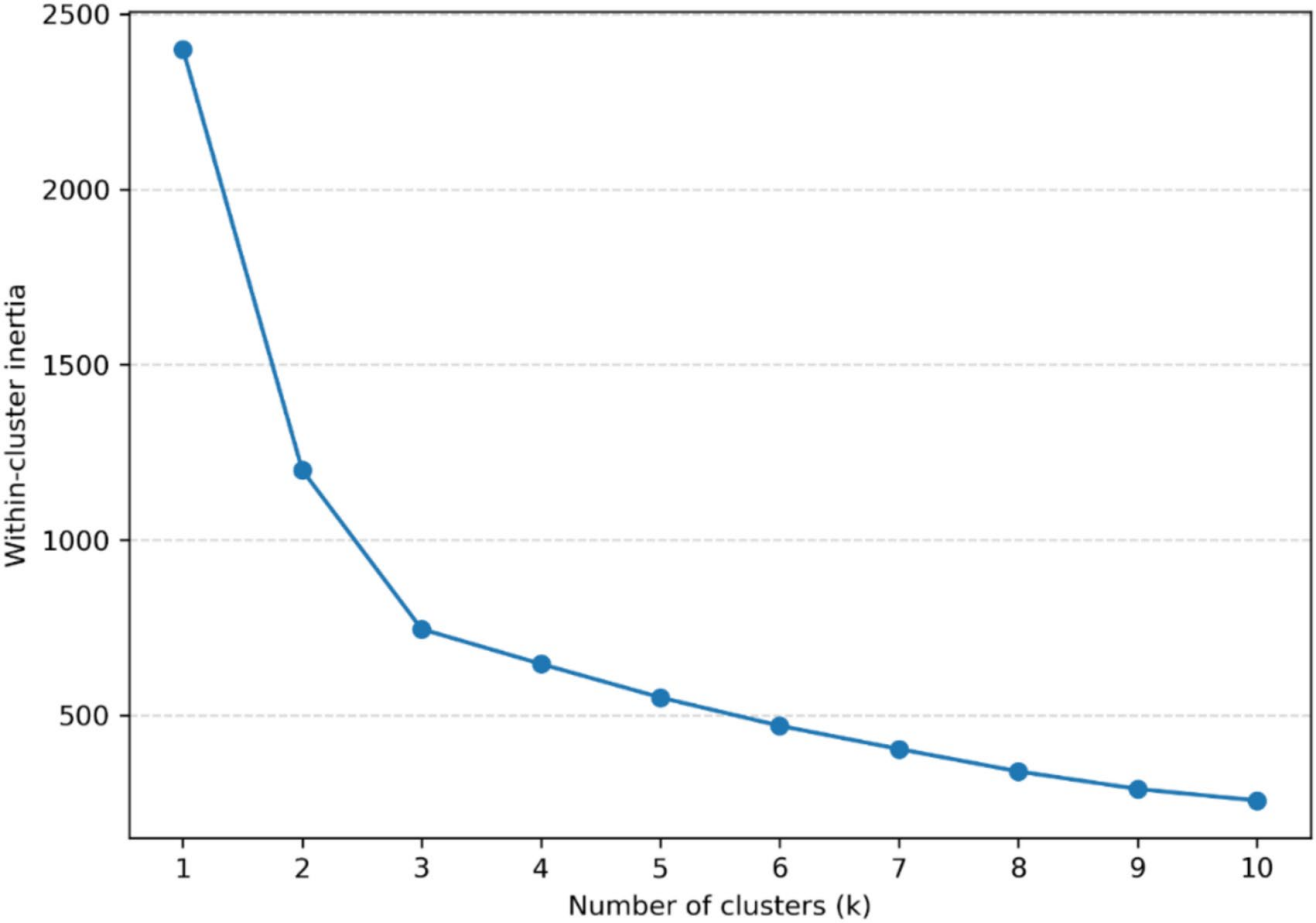
Elbow method used to determine the optimal number of clusters.

An unsupervised learning approach based on k-means clustering was applied to physiological variables, including EEG frequency-band features and ERP-related temporal features such as onset, peak index, peak time, latency, and amplitude. The optimal number of clusters was estimated using the elbow method. The resulting curve suggested a solution between k = 3 and k = 4 clusters, as the reduction in within-cluster inertia becomes progressively less pronounced beyond this range. A solution with k = 4 clusters was selected to provide a more detailed partition of the data while preserving interpretability (see Figure 8).

**Figure 9:**
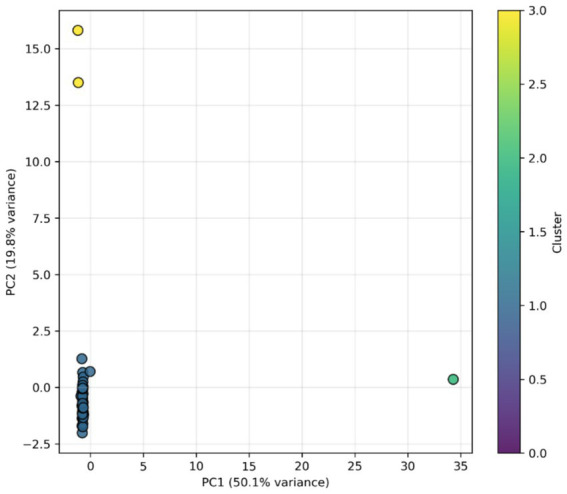
PCA projection of physiological features showing k-means cluster structure (k = 4). The first two principal components explain 50.1 and 19.8% of the variance, respectively. The projection reveals substantial overlap between clusters, with most observations concentrated near the origin of the PCA space, while a small number of points appear more clearly separated, indicating the presence of outliers or distinct physiological response patterns.

Principal Component Analysis (PCA) was employed to reduce data dimensionality and facilitate the visualisation of cluster structure. The projection onto the first two principal components revealed substantial overlap between clusters, indicating limited separability of physiological profiles in the reduced-dimensional space. Most observations were concentrated near the origin, while a small number of points were more dispersed, suggesting the presence of outliers or distinct response patterns. Overall, these results point to weak cluster differentiation, with only partial separation between groups (see Figure 9).

**Figure 10:**
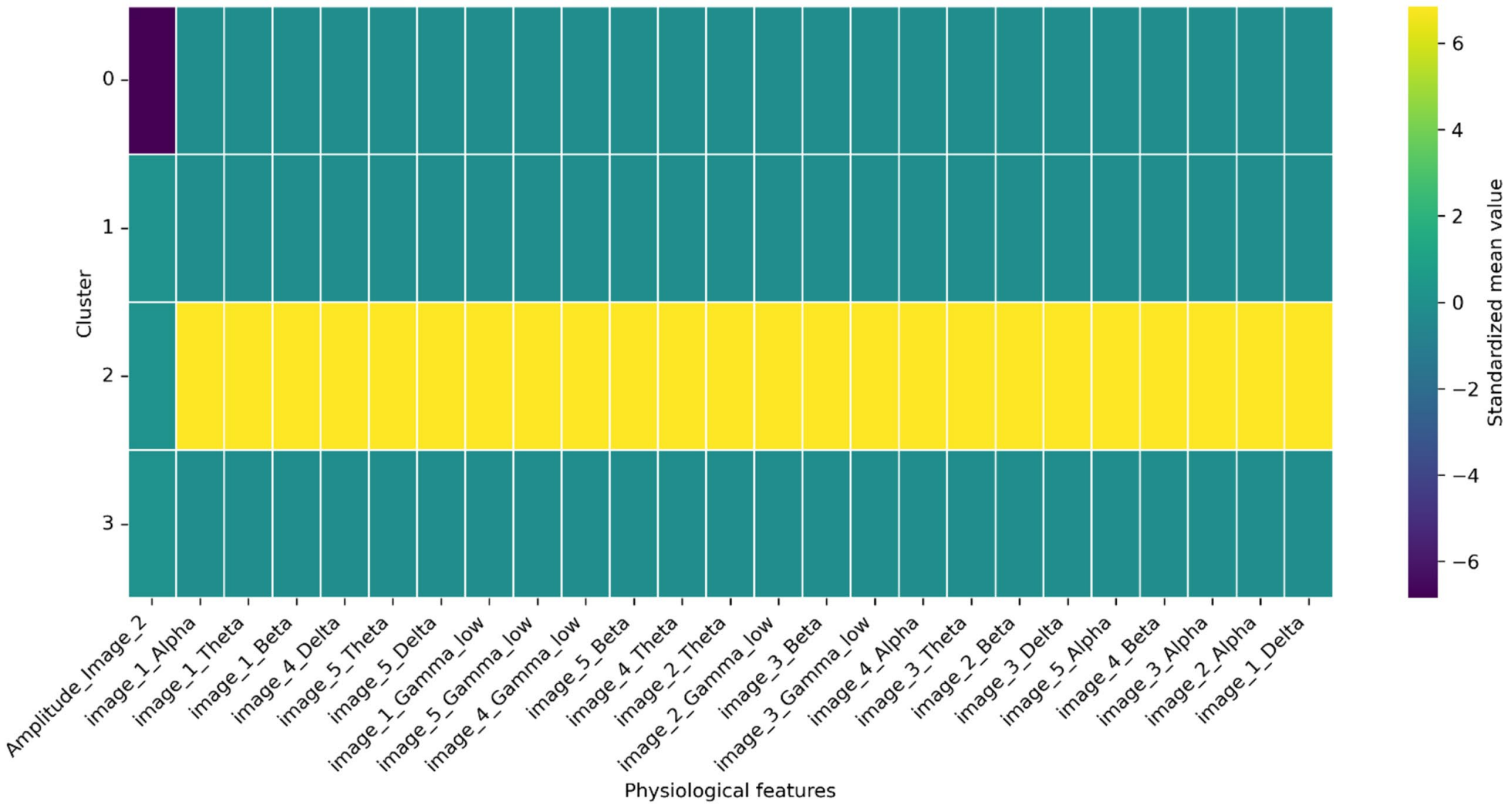
Heatmap of mean physiological feature values across clusters. The colour scale represents standardised feature values, highlighting differences in EEG and ERP-derived variables between clusters.

A heatmap was generated to visualise the mean values of physiological features across clusters (see Figure 10). The results indicate that several temporally derived ERP-related variables—particularly peak time, peak index, onset index, and latency—play a key role in differentiating clusters. These patterns reflect variability in physiological response profiles across participants, with certain clusters characterised by elevated values in specific image-based temporal markers.

**Figure 11:**
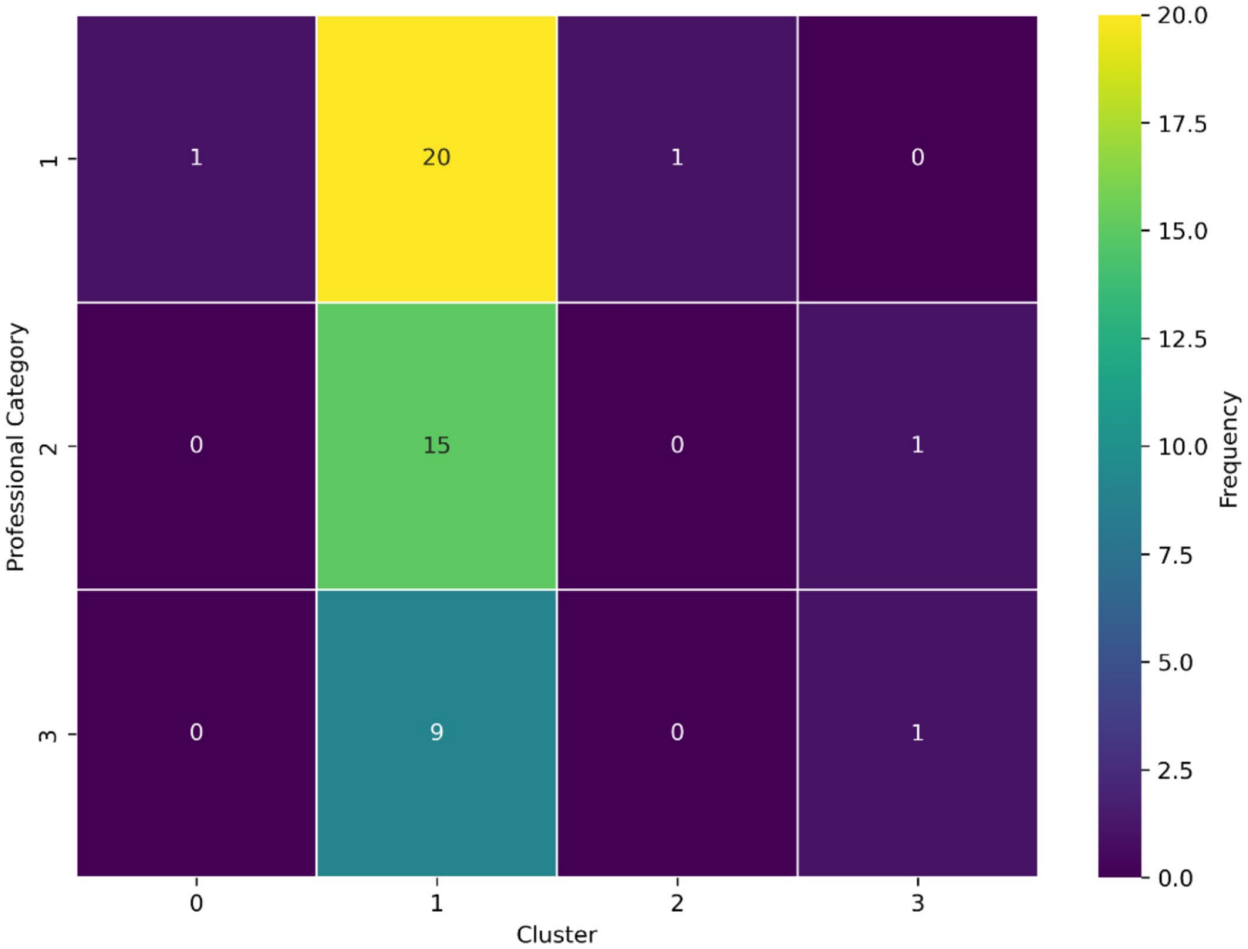
Contingency analysis between professional category and cluster membership. The heatmap shows the frequency distribution of participants across clusters and professional categories. Higher values indicate greater concentration of observations within specific cluster–category combinations.

Subsequently, a contingency analysis was conducted to examine the relationship between professional category and cluster membership derived from the unsupervised learning procedure (see Figure 11). The results showed a contingency coefficient of C = 0.66, indicating a moderate to strong association between the variables. In addition, the chi-square test of independence yielded χ^2^ = 33.27 (*p* = 0.001), confirming that this association was statistically significant.

These findings suggest that cluster membership is not randomly distributed across professional categories, pointing to a partial alignment between the physiological profiles identified through clustering and participant classification.

### 3.4 Dashboard design for automated data collection, processing, and visualisation

The next phase of the study involves the development of a web-based dashboard to support the integrated processing and analysis of multimodal physiological and behavioural data. The system aims to automate key stages of the analytical pipeline—namely preprocessing, modelling, and visualisation—while providing an interactive environment for data exploration and comparison of results.

The proposed dashboard is structured into several functional modules. First, a data acquisition and management module supports the integration and storage of multichannel data, including physiological signals (e.g., EEG- and ERP-derived variables) and associated behavioural measures. Second, an automated preprocessing module incorporates standard procedures such as data cleaning, normalisation, missing value imputation, feature transformation, and dimensionality reduction techniques (e.g., Principal Component Analysis, PCA), thereby ensuring data quality and consistency. Third, an analytical module enables the application of supervised and unsupervised ML techniques, including classification models (e.g., Random Forest), evaluated using performance metrics such as accuracy, balanced accuracy, and macro-averaged F1-score, as well as clustering methods (e.g., k-means) to identify latent structures in physiological data. Finally, a visualisation module provides interactive representations of model outputs, including performance metrics, feature importance, and cluster structure, presented through graphical formats such as bar charts, confusion matrices, heatmaps, dimensionality reduction projections, and contingency analyses, facilitating the interpretation of complex results.

Overall, the dashboard is designed to support exploratory analysis in applied research contexts by enabling the integration of multimodal data and the transparent identification of interpretable patterns.

**Figure 12:**
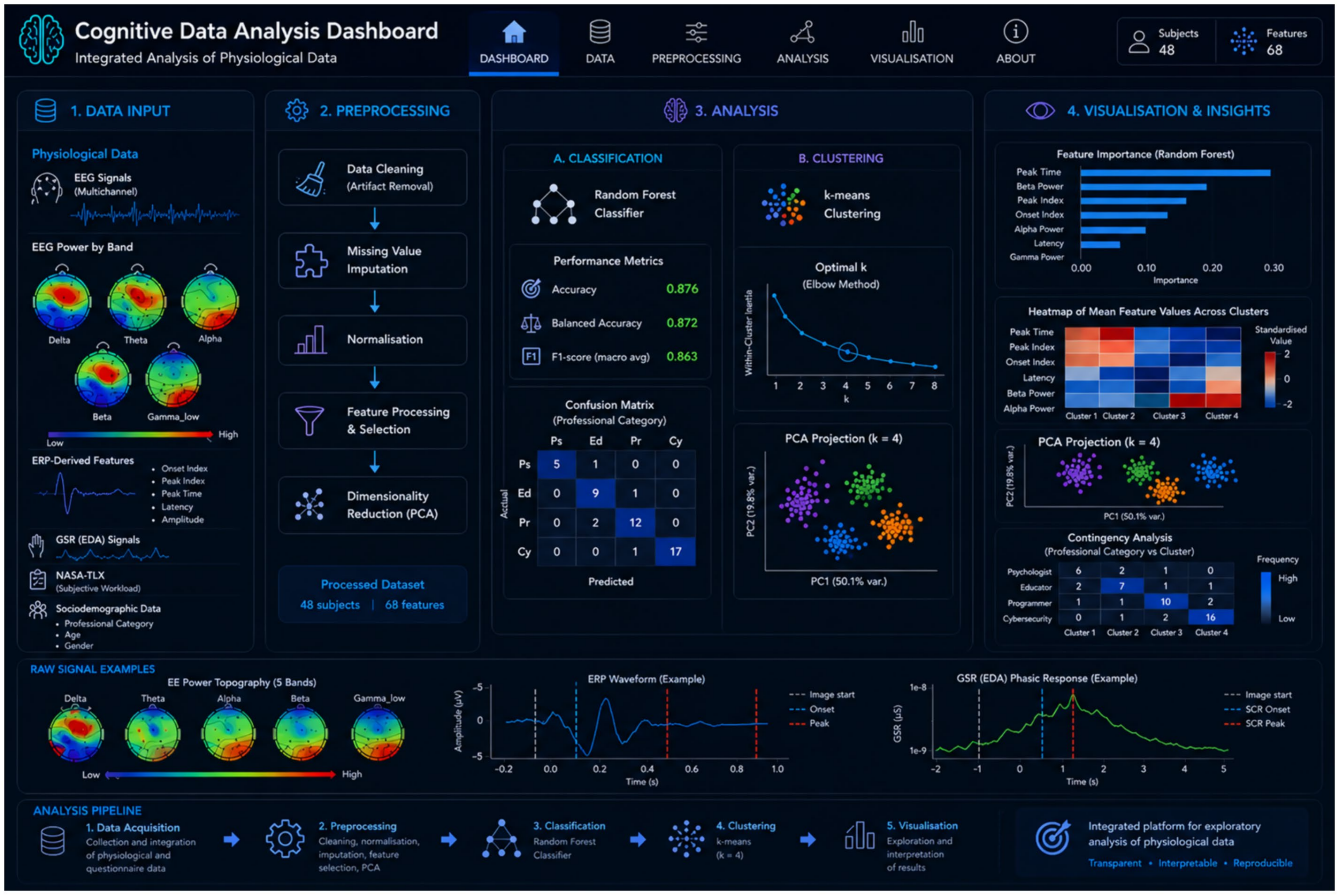
Conceptual representation of the proposed dashboard for the integrated analysis of physiological data. The system includes modules for data preprocessing, classification using Random Forest models, unsupervised clustering (k-means), and visualisation of results, including feature importance, PCA projections, heatmaps, and contingency analysis. The image was generated using our own data and instructions given to an AI (ChatGPT).

**Figure 13:**
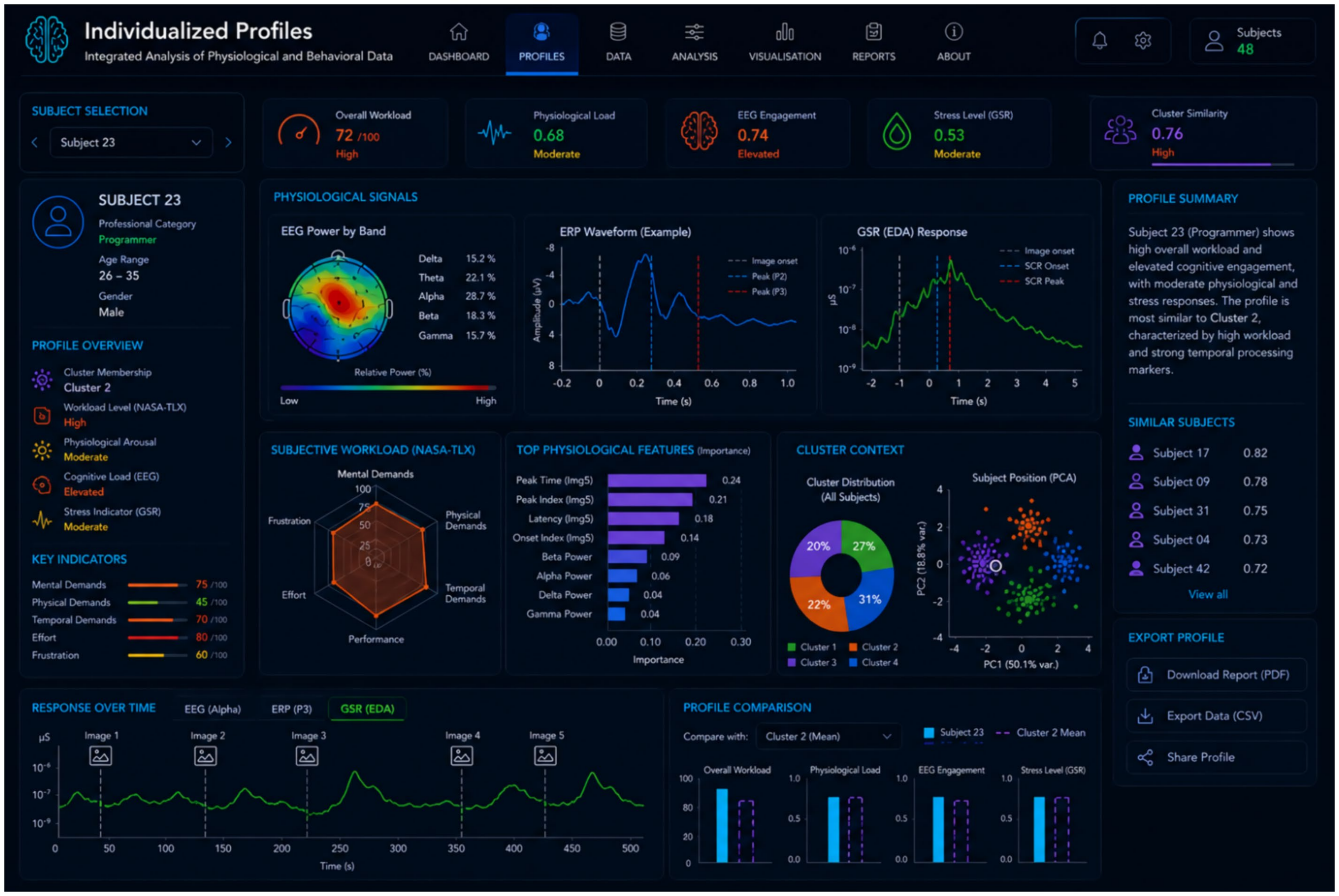
Example of the subject-level analysis module within the proposed dashboard. The interface provides an integrated view of individual physiological, behavioural, and subjective data, including EEG and ERP-derived features, workload indicators (NASA-TLX), feature importance, cluster membership, and comparison with group-level profiles. This functionality enables the identification of individual patterns and supports personalised interpretation and decision-making in applied psychological contexts. The image was generated using our own data and instructions given to an AI (ChatGPT).

Figure 12 presents a conceptual representation of the proposed dashboard for the integrated analysis of multimodal physiological signals. The image was generated using a GAI model and incorporates real processing outputs derived from the present study. Figure 13 further illustrates the subject-level analysis functionality of the dashboard, providing an individualised representation of physiological, behavioural, and contextual data.

## 4 Discussion

The use of integrated multichannel signal recording represents a promising approach in educational psychology for obtaining objective indicators of cognitive and emotional processes. In line with previous research (Cruz-González et al., 2025), the application of AI and ML techniques enables the identification of complex patterns in multimodal physiological and behavioural data, extending beyond traditional self-report measures.

In this context, ML methods provide psychology professionals with tools for pattern recognition, prediction, classification, and the detection of outliers and latent structures through unsupervised approaches. These capabilities are particularly relevant for advancing data-driven assessment, supporting differential diagnosis, and informing the design of personalised intervention programmes (Jones, 2017; Sáiz-Manzanares et al., 2025).

In the present exploratory study, sociodemographic variables showed the greatest predictive power for classifying professional category, outperforming both physiological and subjective workload measures. These results suggest that categorical distinctions among participants are more strongly associated with demographic and contextual factors than with physiological response patterns.

Physiological variables—particularly EEG frequency bands and ERP-derived temporal features—showed limited discriminative capacity in the classification task. This is consistent with the overlap observed in the PCA projection and the relatively low performance of the physiological model. Together, these findings indicate that physiological signals capture meaningful variability, although this variability is not easily separable into discrete categories and is better conceptualised as continuous response profiles.

Subjective workload measures (NASA-TLX) showed intermediate performance, indicating that perceived cognitive load contributes to classification but is less informative than sociodemographic variables. This finding highlights the complementary role of subjective and physiological data in modelling cognitive and emotional processes.

From a methodological perspective, ensemble-based approaches such as the Random Forest classifier proved effective for handling high-dimensional data and identifying relevant predictors, in line with established recommendations (Hastie et al., 2009; James et al., 2021). Clustering results further support this interpretation by revealing partially overlapping groups, reinforcing the view that physiological response patterns are better understood as continuous rather than discrete constructs.

Within the educational context examined in this study, these findings support the potential role of pedagogical avatars as agents of emotional and metacognitive scaffolding. As suggested by previous work (Zheng et al., 2023; Dever et al., 2025; Cloude et al., 2025), adaptive systems informed by physiological data may support the regulation of cognitive load and self-regulated learning processes. In this regard, the proposed dashboard constitutes a tool for integrating multimodal data and facilitating the interpretation of complex patterns in applied educational settings.

### 4.1 Study limitations

Several limitations should be considered when interpreting the results. First, the relatively small sample size limits the generalisability of the findings and increases the risk of overfitting. In addition, the unequal distribution across participant groups may introduce bias and reduce comparability.

Second, the absence of randomisation or counterbalancing may have introduced order effects, such as fatigue or learning bias, which should be considered when interpreting the results. From a methodological perspective, the limited classification performance observed for physiological variables highlights the difficulty of discriminating between categorical profiles based solely on physiological data. This is consistent with the overlap observed in the PCA space and suggests that physiological signals may reflect continuous variability rather than discrete group differences. Furthermore, the absence of inferential statistical testing limits the ability to draw statistically generalisable conclusions. Future studies should incorporate larger samples and hypothesis-driven designs, including inferential analyses such as ANOVA or mixed-effects models. Although a multimodal approach was adopted, the absence of additional physiological signals (e.g., eye-tracking or heart rate variability) may have reduced the explanatory capacity of the models.

In addition, the emotional stimuli used in the study were not subjected to formal psychometric validation to ensure their effectiveness in eliciting specific emotional responses. Consequently, the findings should be interpreted in terms of cognitive and affective processing associated with stimulus perception, rather than as direct evidence of discrete emotion elicitation. Future research should incorporate validated stimulus sets or independent validation procedures.

Finally, this study should be situated within an applied framework. Rather than aiming to develop optimised predictive models, it illustrates the potential of integrating multichannel physiological signal recording with both supervised and unsupervised ML techniques for applied psychology. This approach enables the exploration of cognitive and behavioural patterns, the identification of inter-individual differences, and the detection of latent structures. These functionalities are particularly relevant in educational and clinical contexts, where they may support differential diagnosis and the design of personalised intervention strategies.

### 4.2 Applications in naturalistic environments

The findings of this study highlight the potential for transfer to real-world educational and clinical contexts. The integration of objective physiological signals (EEG and ERP-derived features) with AI and ML techniques enables continuous and non-intrusive analysis of cognitive and emotional processes in ecologically valid settings, helping to address limitations associated with controlled laboratory environments.

In both educational and clinical domains, these approaches support the development of adaptive systems that respond to individuals’ cognitive and emotional states. In Higher Education, this is reflected in intelligent learning environments in which pedagogical avatars act as support agents, facilitating emotional and metacognitive scaffolding and dynamically adapting interactions based on physiological data. This is particularly relevant in digital, blended, and simulation-based learning contexts.

In clinical settings, predictive models based on physiological signals open new possibilities for personalised and preventive interventions. Continuous monitoring may support the early detection of cognitive overload, stress, or emotional dysregulation, enabling adaptive self-regulation strategies and tailored therapeutic approaches.

Interactive dashboards play a key role by enabling the integration, analysis, and real-time visualisation of multimodal data. These tools support informed decision-making across educational and clinical settings, helping to bridge the gap between advanced data analysis and practical application.

Based on these results, an approach to the design of adaptive avatars can be outlined, tailored to the physiological profiles identified through clustering. Using GAI tools, personalised representations can be generated and aligned with observed response patterns. This illustrates a potential application of ML techniques in instructional psychology, contributing to the development of adaptive, data-driven, and person-centred environments.

### 4.3 Future research directions

Based on the findings of this study, several directions for future research can be identified. First, replication studies with larger and more heterogeneous samples are needed to improve external validity and reduce the risk of overfitting. Future work should also incorporate hypothesis-driven designs and inferential statistical analyses (e.g., ANOVA or mixed-effects models) to strengthen the robustness of the findings.

Second, longitudinal designs are required to examine the temporal dynamics of cognitive and emotional processes, as well as the sustained effects of adaptive interventions based on physiological data. This would help determine whether the observed patterns remain stable over time or evolve in response to learning and contextual factors.

Third, the multimodal framework should be extended in future studies by incorporating additional physiological and behavioural data sources, such as eye-tracking, heart rate variability (HRV), or motor activity, which were not included in the present study. The integration of these complementary signals may enhance the explanatory and predictive capacity of ML models and improve the discrimination of user profiles, which showed limited performance under the current multimodal configuration based on EEG and GSR data.

From a methodological perspective, future research should explore and compare different algorithmic approaches, including deep learning models, ensemble methods, and hybrid strategies. Particular attention should be paid to balancing predictive performance and model interpretability, especially in applied educational and clinical contexts. Further work is needed to optimise experimental designs, including the implementation of randomisation and counterbalancing procedures, to minimise potential order effects and improve internal validity.

Regarding model optimisation, hyperparameter tuning was not extensively performed in the present study due to its exploratory nature and limited sample size. Although standard configurations were used to prioritise stability and interpretability, this may have constrained model performance. Future research should therefore incorporate systematic hyperparameter optimisation procedures to improve accuracy and robustness.

Finally, further research should advance the development of ethical and regulatory frameworks governing the use of biometric data, while promoting open science practices that enhance transparency, reproducibility, and cross-context validation of models.

## 5 Conclusion

The application of ML techniques to multimodal physiological data provides a promising framework for advancing the analysis of cognitive and emotional processes in psychological research. In particular, the integration of multichannel physiological signals—such as EEG and ERP-derived features—with behavioural and contextual data enables a more comprehensive characterisation of complex cognitive and affective dynamics than single-modality approaches.

The findings of this exploratory study indicate that, while physiological indicators capture meaningful variability across participants, their discriminative capacity for classifying professional category remains limited when considered in isolation. In contrast, sociodemographic variables emerged as the most informative predictors, with subjective workload measures providing complementary information. These results suggest that physiological signals reflect continuous response patterns rather than clearly separable categorical profiles.

From an analytical perspective, this study highlights the value of combining supervised and unsupervised ML approaches. Classification models support prediction and the identification of relevant predictors, whereas clustering techniques enable the detection of latent structures and partially overlapping physiological profiles without predefined labels. This dual approach facilitates both hypothesis-driven analysis and the identification of emergent patterns, supporting more nuanced interpretations of individual differences. A key contribution of this work is its interdisciplinary perspective, integrating approaches from computer science and biomedical engineering into applied psychological research. Although ML techniques are well established in technical domains, their application in psychology—particularly in real educational environments—remains limited in terms of multimodal signal integration and methodological implementation. This study helps bridge this gap by combining physiological data analysis, ML modelling, and ecologically valid educational data within a unified analytical framework. Overall, the findings provide a proof of concept for the application of AI in authentic psychological and educational settings, highlighting the potential of multimodal and data-driven approaches to support adaptive, personalised, and context-aware practices, while also emphasising the need for further research to improve model robustness, expand multimodal integration, and ensure the ethical and responsible use of biometric data.

From an applied perspective, the integration of AI and ML techniques within dashboard-based systems illustrates how complex multimodal data can be automatically processed, analysed, and visualised in an interpretable manner. This approach provides psychology professionals with tools for pattern detection, prediction, and the identification of latent groupings, supporting differential diagnosis and the design of personalised intervention strategies in educational and clinical contexts.

## Data Availability

The data analyzed in this study is subject to the following licenses/restrictions: the datasets will be made available upon request, accompanied by the accreditation of the requesting institution, a description of the study objectives, and certification from the corresponding institutional ethics committee. Requests to access these datasets should be directed to mcsmanzanares@ubu.es.
