# Supplementary material for: Emotion recognition from multimodal biosignals: supervised and unsupervised machine learning approaches based on EEG and GSR
Source: Front Psychol. 2026 Jul 8;17:1835911. doi: 10.3389/fpsyg.2026.1835911 (PMC13388283; doi:10.3389/fpsyg.2026.1835911)
Supplement: Supplementary file 1 [file Supplementary_File_1.pdf]

Supplementary Material

1 Supplementary Data

This supplementary material provides additional information to support the interpretation of the main results. It includes representative examples of phasic electrodermal activity (EDA) signal processing for one participant from each cluster, as well as a proposal for adaptive avatar profiles derived from the GSR-based cluster analysis.

Figures S1–S4 illustrate representative EDA signal patterns for each cluster. These examples allow visual comparison of differences in phasic activity, including detected onsets and peaks, across the physiological response profiles identified in the clustering analysis.

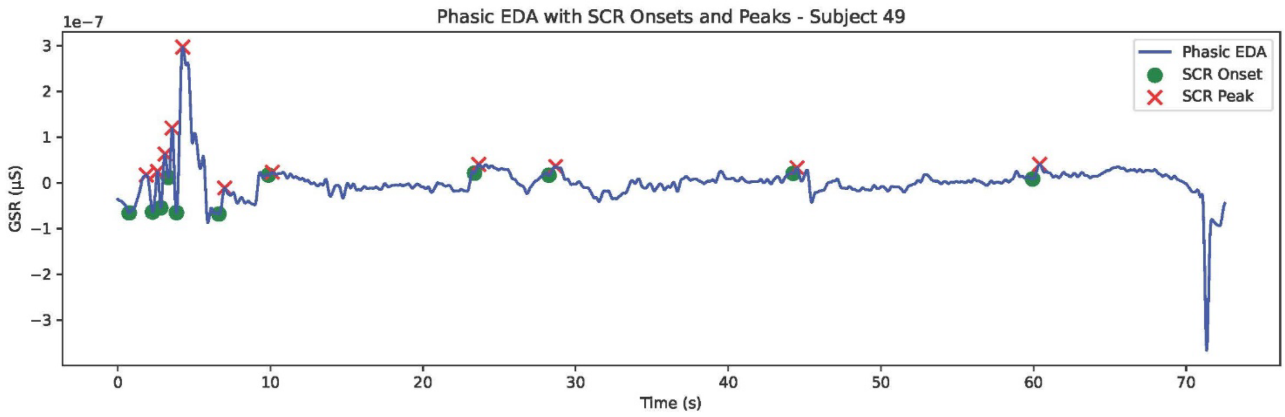

Figure S1. Example of phasic electrodermal activity (EDA) signal with detected onsets and peaks for Subject 49 (Cluster 0). This example illustrates the EDA processing procedure and provides a representative pattern of physiological response for Cluster 0.

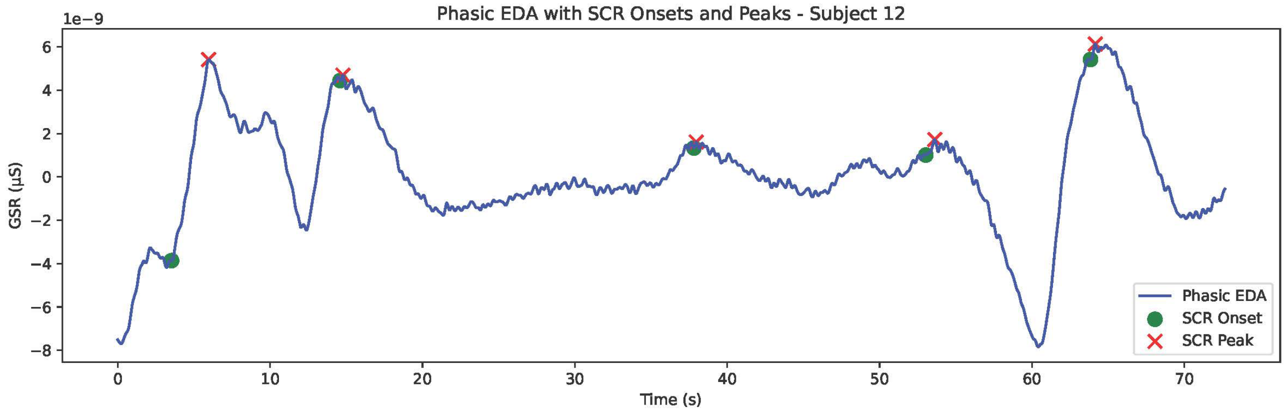

Figure S2. Example of phasic electrodermal activity (EDA) signal with detected onsets and peaks for Subject 12 (Cluster 1). This example illustrates the EDA processing procedure and provides a representative pattern of physiological response for Cluster 1.

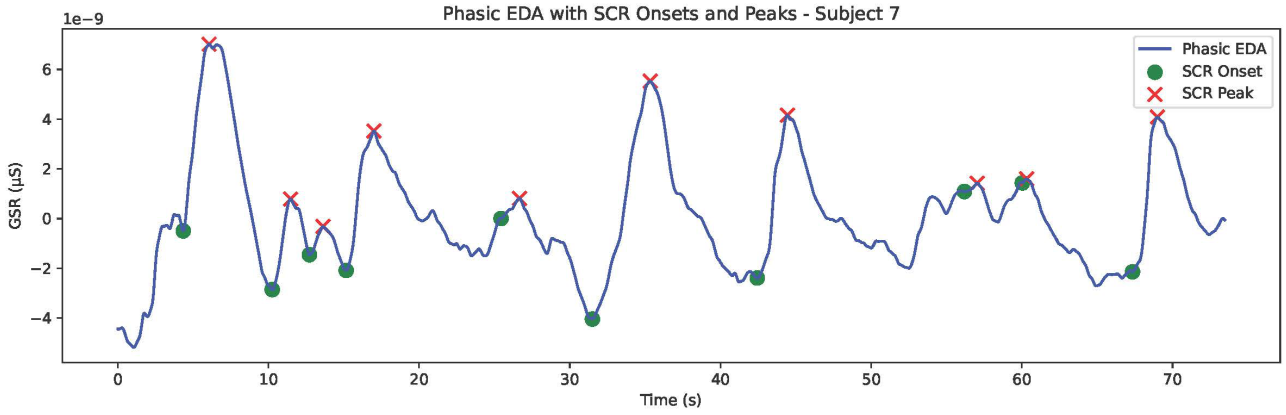

Figure S3. Example of phasic electrodermal activity (EDA) signal with detected onsets and peaks for Subject 7 (Cluster 2). This example illustrates the EDA processing procedure and provides a representative pattern of physiological response for Cluster 2.

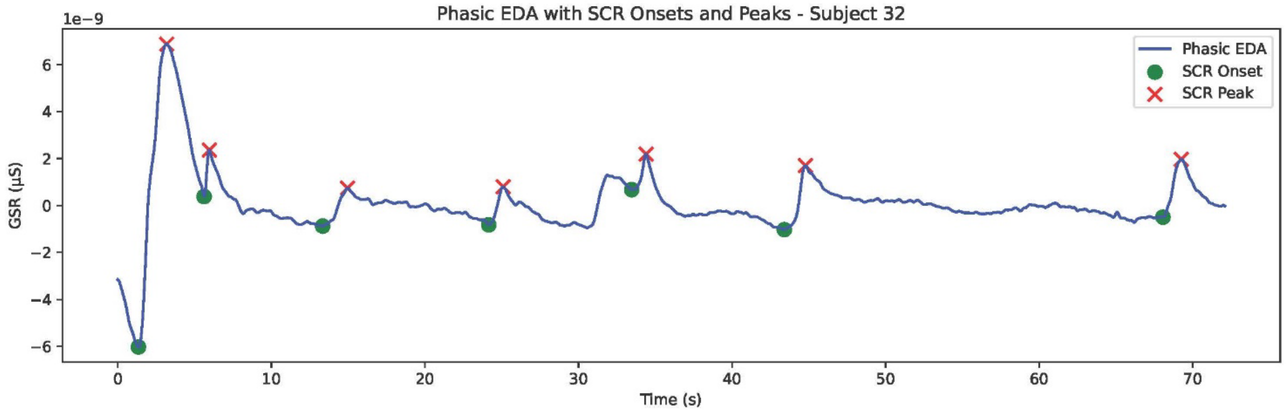

Figure S4. Example of phasic electrodermal activity (EDA) signal with detected onsets and peaks for Subject 32 (Cluster 3). This example illustrates the EDA processing procedure and provides a representative pattern of physiological response for Cluster 3.

Table S1 presents the proposed avatar profiles derived from the GSR-based cluster analysis. Each profile is described according to its main emotional pattern, avatar characteristics, and potential pedagogical application.

Table. S1. Proposed avatar stimuli derived from GSR-based cluster analysis.

Cluster	Objective	Main Emotion	Avatar Characteristics	Pedagogical Application	Example generated using generative AI tools
0	Enhance emotional regulation in response to negative stimuli	Anger and sadness	Calm facial expression, soft gaze, slow movements, low-arousal posture	Emotional regulation training in high-stress learning situations	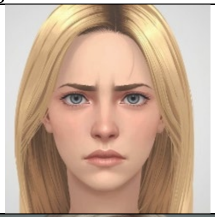
1	Support management of sadness-related states	Sadness	Empathetic expression, slow blinking, slightly lowered gaze, warm tone	Emotional support and reflective learning tasks	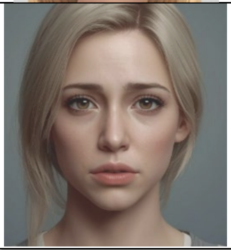
2	Train identification and regulation of anger or frustration	Anger	Alert expression, moderate arousal, focused gaze, controlled gestures	Training in frustration tolerance and impulse control	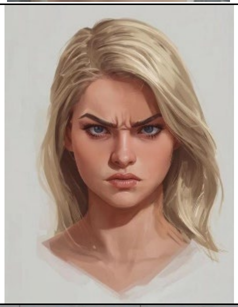
3	Develop emotional flexibility across affective states	Mixed (positive and negative)	Dynamic expressions, adaptive gaze, variable arousal levels	Adaptive learning environments and emotional awareness training	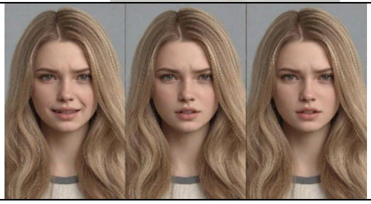

Note. The images were generated using Canva AI as illustrative examples based on the characteristics identified for each cluster. These examples are intended to support the conceptual design of adaptive pedagogical avatars and should not be interpreted as validated intervention materials. Images produced through the authors' own data processing.
